# Factors affecting polyhydroxybutyrate accumulation in mesophyll cells of sugarcane and switchgrass

**DOI:** 10.1186/1472-6750-14-83

**Published:** 2014-09-10

**Authors:** Richard B McQualter, Maria N Somleva, Leigh K Gebbie, Xuemei Li, Lars A Petrasovits, Kristi D Snell, Lars K Nielsen, Stevens M Brumbley

**Affiliations:** 1 Australian Institute for Bioengineering and Nanotechnology, the University of Queensland, Brisbane QLD 4072, Australia; 2 Metabolix, Inc, 21 Erie St, Cambridge, MA 02139, USA; 3 Current address: Agrivida, 200 Boston Ave, Medford, MA 02155, USA; 4 Current address: Department of Biological Sciences, The University of North Texas, 1155 Union Circle #305220, Denton, TX 76203-5017, USA

**Keywords:** Polyhydroxybutyrate, Sugarcane, Switchgrass, Biomass, C4 grasses, Acetyl-CoA, Biopolymer

## Abstract

**Background:**

Polyhydroxyalkanoates are linear biodegradable polyesters produced by bacteria as a carbon store and used to produce a range of bioplastics. Widespread polyhydroxyalkanoate production in C_4_ crops would decrease petroleum dependency by producing a renewable supply of biodegradable plastics along with residual biomass that could be converted into biofuels or energy. Increasing yields to commercial levels in biomass crops however remains a challenge. Previously, lower accumulation levels of the short side chain polyhydroxyalkanoate, polyhydroxybutyrate (PHB), were observed in the chloroplasts of mesophyll (M) cells compared to bundle sheath (BS) cells in transgenic maize (*Zea mays*), sugarcane (*Saccharum* sp.), and switchgrass (*Panicum virgatum* L.) leading to a significant decrease in the theoretical yield potential. Here we explore various factors which might affect polymer accumulation in mesophyll cells, including targeting of the PHB pathway enzymes to the mesophyll plastid and their access to substrate.

**Results:**

The small subunit of Rubisco from pea effectively targeted the PHB biosynthesis enzymes to both M and BS chloroplasts of sugarcane and switchgrass. PHB enzyme activity was retained following targeting to M plastids and was equivalent to that found in the BS plastids. Leaf total fatty acid content was not affected by PHB production. However, when fatty acid synthesis was chemically inhibited, polymer accumulated in M cells.

**Conclusions:**

In this study, we provide evidence that access to substrate and neither poor targeting nor insufficient activity of the PHB biosynthetic enzymes may be the limiting factor for polymer production in mesophyll chloroplasts of C_4_ plants.

## Background

C_4_ photosynthesis is more complex than the ancestral C_3_ pathway of carbon fixation and involves morphological and biochemical modifications leading to a significant reduction in photorespiration. This is achieved by compartmentalization of the processes of carbon dioxide (CO_2_) fixation and reduction between physiologically distinct mesophyll (M) and bundle sheath (BS) cells. The resulting increase in the photosynthesis rate allows C_4_ plants to achieve high biomass yields under adverse conditions, such as drought, high temperature, and CO_2_ or nitrogen limitation. It also favours their use for the production of biofuels [1,2] and as ‘green’ chemical biofactories [3-5] to produce replacements for petroleum-derived products.

Polyhydroxyalkanoates (PHAs), linear polyesters produced naturally in bacteria as a means of storing carbon [6,7], are an ideal molecule to produce in C_4_ plants. When extracted and processed they have similar properties to many common plastics but are fully biodegradable. Large-scale production of PHAs in high biomass yielding crops would provide a sustainable supply of bioplastics and energy from one plant feedstock that could be processed economically in a biorefinery [8]. The simplest PHA, polyhydroxybutyrate (PHB), has been successfully produced in a number of plant species [5,8-11] and has potential applications not only as a bioplastic, but also for the production of chemicals and improved animal feeds [11]. In plants, PHB is produced by the heterologous expression of three bacterial genes encoding a β-ketothiolase (PhaA), an acetoacetyl-CoA reductase (PhaB) and a PHB polymerase (PhaC) [7]. The ketothiolase condenses two molecules of acetyl-CoA to form acetoacetyl-CoA which is reduced to 3-hydroxybutyryl-CoA by the acetoacetyl-CoA reductase. PHB polymerase then incorporates 3-hydroxybutyryl-CoA into the growing polymer chain. Thus, appropriate targeting of the PHB biosynthesis enzymes to subcellular compartments where sufficient acetyl-CoA and reducing power are available is necessary.

In C_4_ plants, PHB has been successfully produced in the plastids [12-15] and peroxisomes [16,17]. Although PHB accumulation in the chloroplasts of the C_4_ crops maize [14] (*Zea mays* L., NADP-ME C_4_ subtype), sugarcane [12,13] (*Saccharum sp*., NADP-ME C_4_ subtype) and switchgrass [11,15] (*Panicum virgatum* L., NAD-ME C_4_ subtype) has reached more than 5% of leaf dry weight (DW) in individual leaves, these levels are not yet commercially viable [11]. The highest PHB levels to date have been reported in chloroplasts of C_3_ plants upon nuclear expression of plastid-targeted enzymes in *Arabidopsis thaliana*[18] (up to ~40% DW) or direct expression of transgenes from the plastome in tobacco [19] (up to 18% DW). Thus the compartmentalization of C_4_ photosynthesis between two distinct cell types, i.e. M and BS cells appears to affect polymer production in high yielding C_4_ biomass crops. In maize, sugarcane and switchgrass [12-15] less PHB is produced in the chloroplasts of M cells than in BS cells. While the cause of this disparity in product accumulation between cell types is unknown, this presents a significant loss in yield potential for PHB in these C_4_ plants. Mesophyll cells occupy on average 79% and 66% of the total chlorenchyma area per vein for the C_4_ subtypes NADP-dependent malic enzyme (NADP-ME) and NAD-dependent malic enzyme (NAD-ME), respectively [20]. Significant increases in PHB production are thus theoretically possible if polymer levels in chloroplasts of M cells can be elevated.

The present study explored the distribution of PHB production between M and BS cells in both sugarcane and switchgrass. We also measured some key metabolites to determine if further insight could be gained into the low M production of PHB. We examined the effect of polymer synthesis on the content of fatty acids, starch and soluble sugars (sucrose and glucose). While *de novo* fatty acid biosynthesis occurs in both types of photosynthetic cells in C4 plants [21] starch is produced preferentially in BS where the Benson-Calvin cycle is localized [22]. The cellular localization of sucrose synthesis varies between C_4_ grasses [23-26].

## Results

### Plant material

Sugarcane lines identified in our previous reports are relabelled as follows with PHB values reported earlier [12,13,15] as the mean ± standard error: Wild-type Q117 (WT), TA4 (LP, 0.45 ± 0.02% DW PHB), 4 F1 (MP1, 1.21 ± 0.24% DW PHB), 8C8 (MP2, 1.30 ± 0.11% DW PHB) and 7C3 (HP, 3.11 ± 0.31% DW PHB). These PHB values reflect data measured in whole-leaf blades from the oldest green leaf. The expression of genes encoding PHB pathway enzymes PhaA, PhaB and PhaC in sugarcane is driven by the maize ubiquitin promoter in LP, the rice ubiquitin promoter in MP1, and the maize *Cab-m5* promoter in MP2 and HP. Sugarcane plants were grown from cuttings taken from the original transgenic plant material and grown in a physical containment glasshouse under ambient lighting conditions. Switchgrass lines identified as WT, LP (0.60 ± 0.08% DW PHB, MP1 (1.10 ± 0.12% DW PHB), MP2 (1.60 ± 0.01% DW PHB), HP (2.44 ± 0.43% DW PHB) represent independent transformation events expressing the PHB biosynthesis genes *phaA*, *phaB* and *phaC*, under the control of the maize *cab-m5* promoter [15]. These PHB values reflect data measured in segments approximately 15 cm from the tip of leaf blades adjacent to the node at the base of the stem. Switchgrass plants were grown in a glasshouse at 28°C with supplemental lighting (16 h photoperiod, sodium halide lamps) and maintained by repotting every six months.

### Distribution of PHB enzymes between M and BS chloroplasts

**Figure 1:**
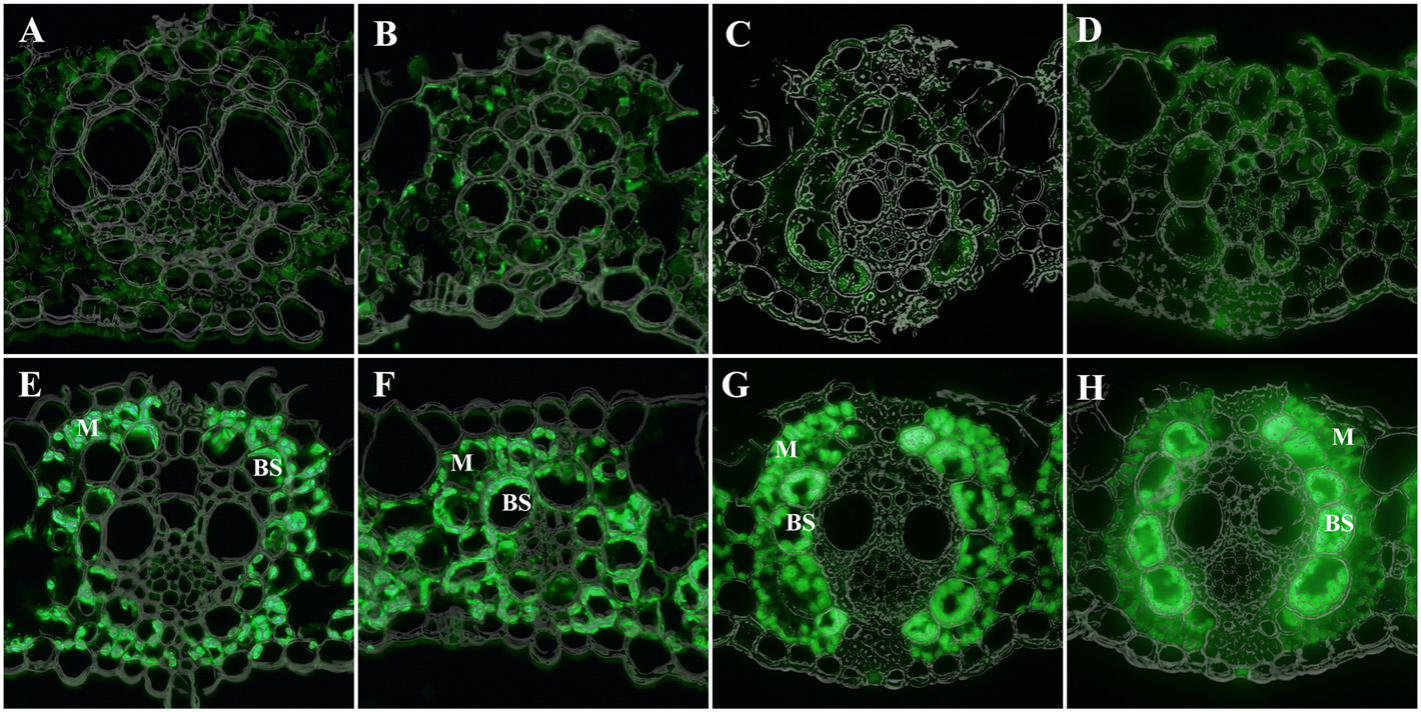
**Transverse sections of sugarcane and switchgrass leaves immunolabelled for PHB biosynthesis proteins PhaA and PhaB.** Wild type sugarcane **(A, B)**; wild type switchgrass **(C, D)**; high PHB-producing sugarcane **(E, F)** high PHB-producing switchgrass **(G, H)**; PhaA **(A, C, E, G)**; PhaB **(B, D, F, H)**.

Immunolocalization of PhaA and PhaB in transverse leaf sections of sugarcane and switchgrass showed that these two proteins are targeted to the chloroplasts of both M and BS cells (Figure 1). Our highest producing sugarcane plants and all of the switchgrass plants contain a hybrid PhaC [27,28] for which antiserum was not available and so immunolocalization of PhaC was not conducted.

### Activity of PHB enzymes in M and BS

**Figure 2:**
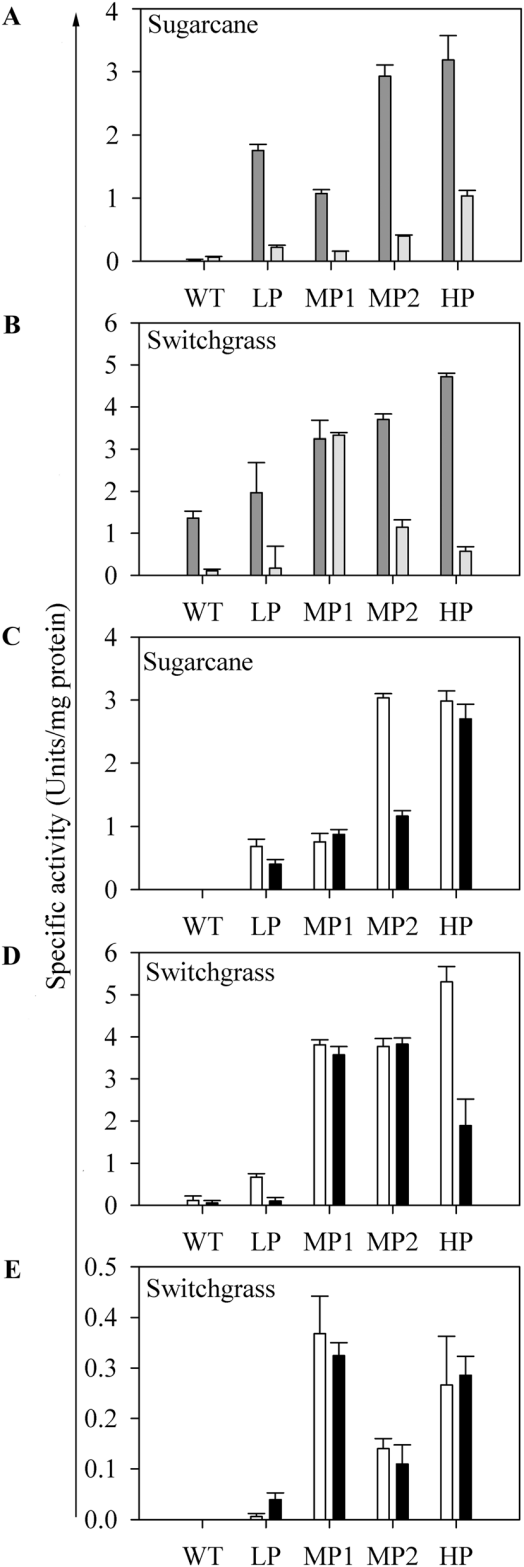
**Enzyme activities in total protein extracts from PHB producing transgenic sugarcane and switchgrass.** A-B PhaA (dark bars) and PhaB (light bars) activity in whole leaf protein extracts of sugarcane **(A)** and switchgrass **(B)**. **(C, D)** PhaA activity in sugarcane **(C)** and switchgrass **(D)** M (white bars) and BS (black bars) chloroplast protein fractions. **(E)** PhaB activity in switchgrass M and BS chloroplast protein fractions. LP, low PHB producer (≤1% DW); MP, medium producer (>1% ≤ 2% DW); HP, high producer (>2% DW). Error bars represent standard error (n = 3).

Enzyme activities were measured in total protein extracted from whole leaf and from M and BS chloroplast fractions. Both PhaA and PhaB activity could be detected in the whole leaf extracts from sugarcane (Figure 2A) and switchgrass (Figure 2B). In all samples analysed, PhaA (Figure 2C & D) and PhaB (Figure 2E) activities were either equal or higher in M than BS chloroplasts. Detection of PhaB activity in sugarcane BS protein fractions did not yield consistent results. This could be due to the instability of the PhaB enzyme in crude extracts during prolonged and difficult procedures to isolate chloroplasts from BS strands.

### Effects on fatty acid synthesis (FAS)

**Figure 3:**
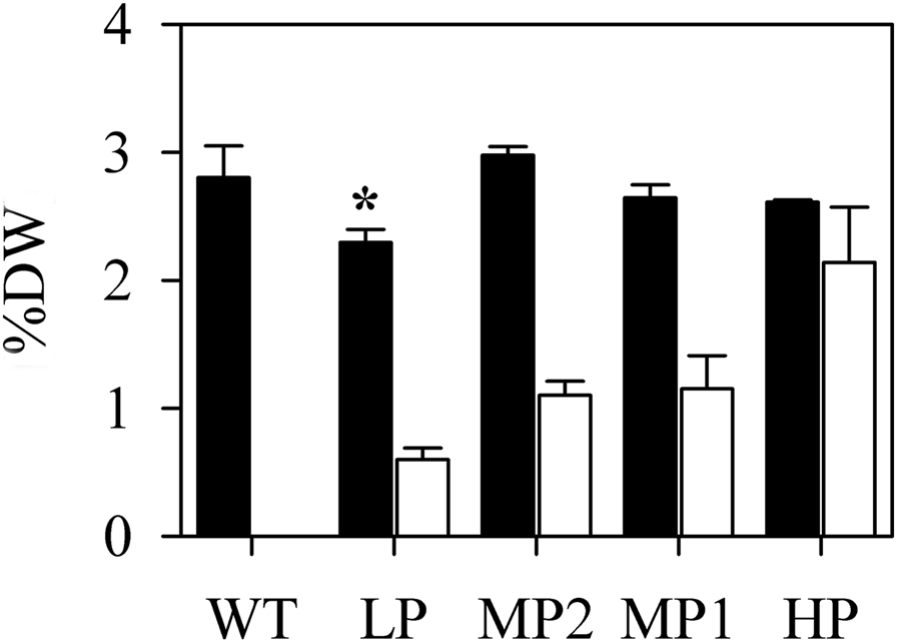
**Total fatty acid content and PHB levels in transgenic and wild type switchgrass plants.** Bars: black, total fatty acids; white, PHB. Values are presented as a percentage of leaf dry weight (DW). Error is standard deviation of the mean, WT, wild type (n = 3); LP, low producer, ≤1% DW (n = 3); MP1 and MP2, medium producers, >1% ≤ 2% DW (n = 3) and HP, high producer, >2% DW (n = 6). *Indicates total fatty acid content significantly different to the wild-type control (P < 0.01).

The observed correct targeting of PhaA and PhaB enzymes to M and BS chloroplasts prompted additional studies to examine differences in carbon availability between the two cell types for PHB synthesis. Plastids are the site of FAS, a process that consumes acetyl-CoA as substrate. Acetyl-CoA carboxylase (ACCase) catalyses the first committed step in FAS by converting acetyl-CoA to malonyl-CoA and PhaA would therefore compete with it for the common substrate.To determine if PHB production affects FAS in our transgenic lines, the total fatty acid content was measured in leaves of low (LP), medium (MP) and high polymer-producing (HP) and wild type switchgrass plants grown in soil for two months. No statistically significant differences in total fatty acid content were observed between the wild type and transgenic lines HP, MP1 and MP2 (Figure 3). The leaves of transgenic line LP, however, contained slightly less fatty acids (P = 0.006) than the other samples. This may be due to a position effect of the transgene insertion since all the transgenic lines analysed in the present study are independent transformation events.

**Figure 4:**
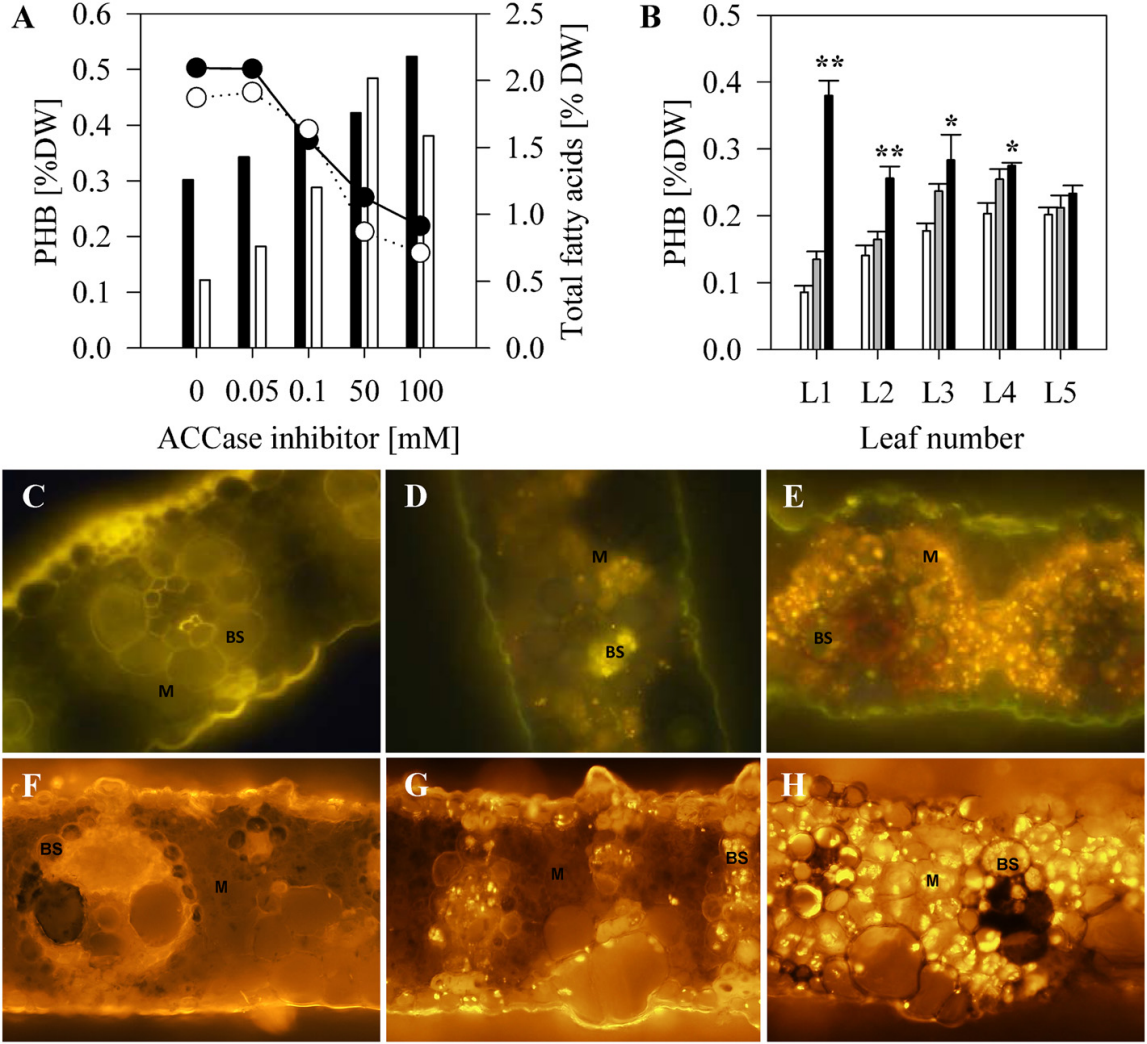
**The effect of ACCase inhibition on PHB production in transgenic switchgrass and sugarcane. (A)** PHB (bars) and total fatty acid (lines) content in 30 leaves of *in vitro* propagated switchgrass plantlets treated with various concentrations of the ACCase inhibitor quizalofop. Shaded and clear bars or circles represent two independent transgenic events. **(B)** PHB content in leaves of increasing maturity (L1 is the youngest leaf and L5 is the most mature one) from soil grown sugarcane plants treated with various concentrations of the ACCase inhibitor fluazifop-p-butyl (Bars: white = 0 μM, grey = 20 μM, black = 100 μM). PHB levels which are significantly different to the untreated control are indicated: **P < 0.001; *P < 0.05). **C**, **D**, **E** Nile Blue A stained leaf sections from *in vitro* propagated switchgrass plants: **C**, WT; **D**, untreated PHB producing plant; **E**, PHB producing plant treated with 100 μM quizalofop. **F**, **G**, **H**, Nile Blue A stained leaf sections from soil-grown sugarcane plants treated with: **F**, WT; **G**, untreated PHB producing plant; **H**, PHB producing plant treated with 100 μM fluazifop. Note the abundance of PHB granules in the M tissue in the Nile Blue A stained sections from treated switchgrass and sugarcane PHB producers.

We have shown previously that inhibition of fatty acid synthesis results in increased production of PHB in sugarcane [29] but did not determine if this was due to increased production in M chloroplasts. To gain an understanding of how this affects the distribution of the polymer between BS and M, switchgrass plants in tissue culture were treated with quizalofop, a herbicide from the aryloxyphenoxypropionate group which inhibits ACCase activity [30,31]. The plants were regenerated from immature inflorescence-derived callus cultures initiated from two high producers, one of which was also used for other experiments in this study (Figures 1 & 2; HP). A concentration-dependent increase in polymer levels accompanied by a reduction in the content of total fatty acids (≥50%) in plants treated with the highest quizalofop concentrations tested) was measured in the herbicide-treated plants compared to the non-treated plants from both lines (Figure 4A). A pronounced increase in the abundance of PHB granules was observed in both BS and M cells of treated leaves as visualized by Nile Blue A staining (Figure 4D, E).

Possible differences in the effect of inhibiting FAS on polymer production in developing leaf tissue, where demand for fatty acids is high [32], and in mature leaf tissue, where the highest PHB levels are measured [15,33], were also investigated. Whole sugarcane leaves of line LP at increasing stages of maturity, starting from the 1st fully unfurled leaf with visible dewlap down to the fifth and oldest leaf (L1-L5), were analysed for PHB content two weeks post-treatment with fluazifop-p-butyl, a structural analogue of quizalofop. In the non-treated transgenic plants, PHB content was lowest in the youngest leaves and increased with leaf age until L4, while in L5 no significant increase in PHB was observed (Figure 4B). Fluazifop-p-butyl applied at a concentration of 100 μM caused a significant increase in PHB content in L1 and L2 (P < 0.001), L3 (P = 0.016), and L4 (P = 0.021) with the largest increases in PHB accumulation observed in the younger leaves (Figure 4B). PHB content increased in L1 from 0.09 ± 0.01 to 0.38 ± 0.02% DW, a 4.4 fold increase. The polymer content increased 1.8, 1.6, and 1.4 fold in L2, L3, and L4, respectively. The increase in PHB in L5, the oldest leaf, from 0.20 ± 0.01 to 0.23 ± 0.01% DW was not statistically significant. As in switchgrass, the abundance of PHB granules increased in both BS and M cells in treated sugarcane leaves (Figure 4G, H).

### Starch and sucrose accumulation

**Figure 5:**
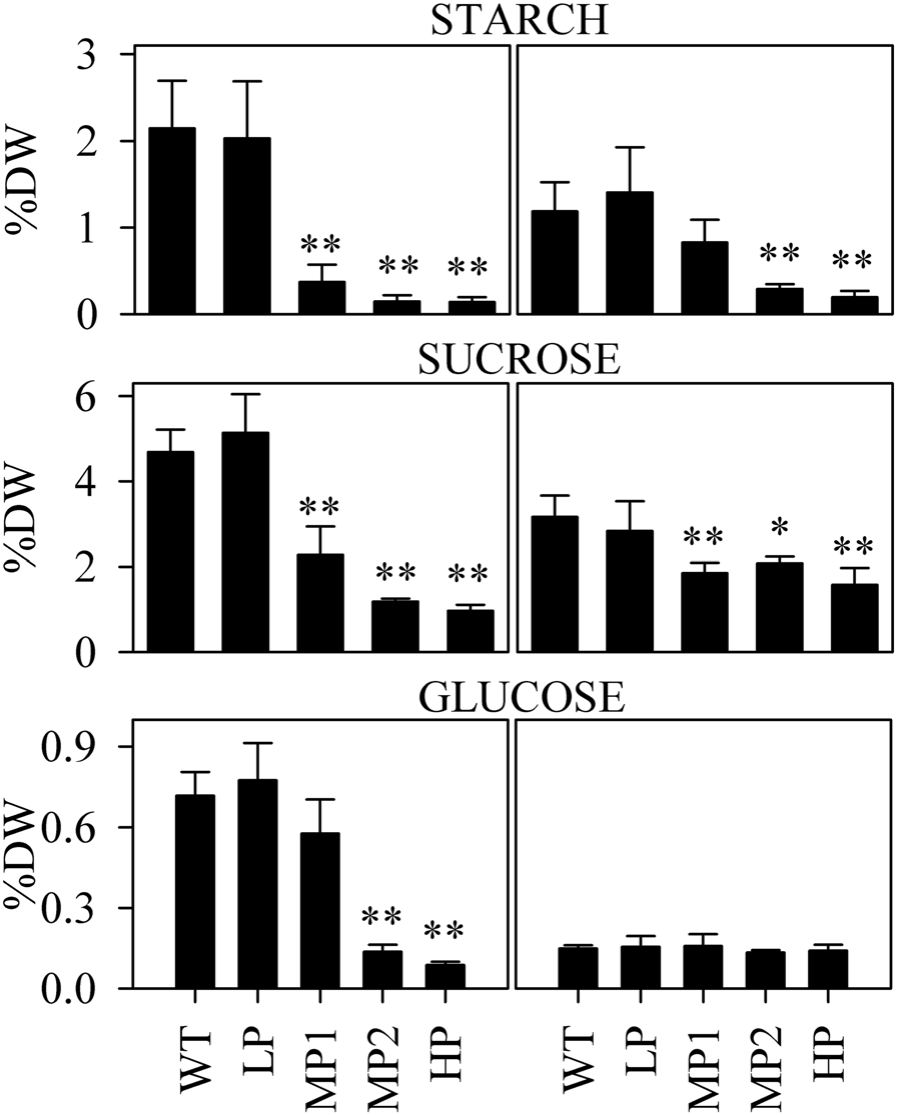
**Effect of PHB production on starch, sucrose and glucose content in sugarcane and switchgrass leaves.** Values are presented as a percentage of leaf dry weight. Error bars show standard deviation (n = 4). Left panels, sugarcane; Right panels, switchgrass. WT, wild type; LP, low PHB producer (≤1% DW); MP, medium producer (>1%, ≤2% DW); HP, high producer (>2% DW). Significant differences are indicated: **P < 0.001; *P < 0.05.

The previously observed lack of starch granules in the BS plastids of polymer producing switchgrass as visualized by TEM [15] suggested that PHB biosynthesis causes a significant reduction in starch accumulation. In this study, starch, sucrose, and glucose contents were measured in four month old PHB producing switchgrass and sugarcane plants (Table 1). In general, both the starch and sucrose levels measured in leaves at approximately midday were inversely correlated to PHB accumulation in transgenic lines (Figure 5). While the content of these two carbohydrates was unaffected in the lowest PHB-producing lines, it was significantly reduced in lines accumulating polymer above 1% DW. In sugarcane leaves, the sucrose content dropped from approximately 5% leaf DW in wild type to about 1% DW in the high PHB-producing lines. Starch levels in these plants decreased by as much as ten-fold. In switchgrass, sucrose levels decreased from 3% DW to 1.5% DW and starch content showed a four-fold decrease in the highest PHB-producing line compared to the wild-type control. In sugarcane, glucose also dropped significantly with increased PHB production whereas glucose content in switchgrass leaves was not significantly affected by PHB production. The sugarcane line MP1 and the switchgrass medium producers MP1 and MP2 with significantly lower sugar and starch contents were similar to wild type plants in terms of leaf chlorophyll and carotenoid contents and biomass yield (results not shown).

**Table 1:** Starch, sucrose and glucose content in the leaves of wild-type and PHB-producing sugarcane and switchgrass plants

**Species**	**ID**	**Starch (±SD)**	**Sucrose (±SD)**	**Glucose (±SD)**
Switchgrass	WT	1.19 ± 0.34	3.16 ± 0.50	0.15 ± 0.01
	LP	1.40 ± 0.52	2.83 ± 0.70	0.16 ± 0.04
	MP1	0.83 ± 0.26	1.85 ± 0.24	0.16 ± 0.04
	MP2	0.29 ± 0.06	2.08 ± 0.15	0.13 ± 0.01
	HP	0.20 ± 0.07	1.58 ± 0.39	0.14 ± 0.02
Sugarcane	WT	2.15 ± 0.54	4.68 ± 0.53	0.72 ± 0.09
	LP	2.03 ± 0.65	5.14 ± 0.91	0.77 ± 0.14
	MP1	0.37 ± 0.20	2.27 ± 0.66	0.58 ± 0.13
	MP2	0.15 ± 0.07	1.18 ± 0.07	0.14 ± 0.03
	HP	0.14 ± 0.06	0.97 ± 0.14	0.09 ± 0.01

Values are presented as a percentage of leaf dry weight ± standard deviation (n = 4). WT, wild type; LP, low producer (PHB ≤1% DW); MP, medium producer (PHB >1%, ≤2% DW); HP, high producer (PHB >2% DW).

## Discussion

### PHB enzymes are equally abundant and active in M and BS cells

In our first study of plastid-targeted PHB production in sugarcane, where the PHB pathway genes were all driven by the maize ubiquitin promoter, both fluorescent staining patterns and TEMs demonstrated the lack of PHB accumulation in M chloroplasts [12]. This cell specific pattern of polymer accumulation appears to be promoter independent as it did not occur when PHA production was targeted to the peroxisomes using the same promoter [17]. More recently, we used the maize *cab-m5* promoter to express the PHB genes in switchgrass and sugarcane and although some PHB accumulated in M plastids, it was much less than the polymer detected in BS cells [13-15]. Differential expression of transgenes in M and BS cells is not thought to contribute to this disparity in product accumulation [15] since the *cab-m5* gene is known to be expressed in both types of cells in maize yielding more abundant transcripts in M cells [34]. Greater PHB production in BS chloroplasts has also been observed with the rice ubiquitin 2 promoter [35] in both switchgrass [15] and sugarcane [13]. In the present study, we have confirmed that the signal peptide of the small subunit of Rubisco from pea effectively targets the PHB biosynthesis enzymes to both M and BS chloroplasts of sugarcane and switchgrass (Figure 1) as previously shown in maize [36]. Enzyme assays also showed that PHB enzyme activity is retained following targeting to M plastids and that it is equivalent to that found in the BS plastids (Figure 2). Together these results suggest that anomalous targeting of the PHB biosynthesis enzymes to M plastids is not a contributing factor to low levels of polymer accumulation in this cell type as previously speculated [33].

### Diversion of acetyl-CoA to PHB does not affect total fatty acid content

In chloroplasts, PHB biosynthesis competes directly with *de novo* fatty acid biosynthesis for substrate since both pathways use acetyl-CoA. Despite this competition, PHB production in in switchgrass leaves did not reduce the total fatty acid content (Figure 3). This has also been observed in transgenic *Arabidopsis* where substantial production of PHB up to ~40% DW resulted in no net changes in total fatty acids [18]. Nawrath et al. [37] reported that high polymer producing *Arabidopsis* plants (~14% DW) grew normally leading the authors to speculate that a significant increase in the net flux through the plastidic acetyl-CoA pool might occur in response to the introduction of a PHB carbon sink and that mechanisms might exist to enhance synthesis of acetyl-CoA in response to demand.

### Inhibition of fatty acid synthesis enhances PHB biosynthesis in M cells

Acetyl-CoA carboxylase (ACCase), which catalyses the first committed step of *de novo* fatty acid biosynthesis and thus is the major flux-controlling enzyme of the pathway [38], competes directly with the thiolase for acetyl-CoA. Chemical inhibition of ACCase has previously been shown to increase PHB production in both C_3_[39] and C_4_ plants [29]. When fatty acid biosynthesis was inhibited in developing sugarcane leaves, PHB production increased four-fold (Figure 4B) indicating that substantial flux can be redirected to polymer synthesis. A significant increase in polymer accumulation was most apparent in M chloroplasts as visualized by fluorescence microscopy, but was also detected in BS chloroplasts in both sugarcane and switchgrass (Figure 4E, H). This supports our hypothesis that access to substrate is the main contributing factor to low levels of PHB accumulation in mesophyll chloroplasts.

We found that the rate of polymer accumulation in the leaves of non-treated PHB producing sugarcane is greatest during leaf maturation and plateaus as the leaf completes development (Figure 4B). The state of leaf maturity also affects interaction between fatty acid and PHB biosynthetic pathways. In developing leaves, a large flux of carbon through fatty acid synthesis supports the formation and elongation of new cells [32]. Indeed, ACCase protein abundance was recently shown to peak sharply in the elongating zone of the developing maize leaf, where maximum rates of fatty acid synthesis are required to support plant growth [26]. Inhibition of this pathway during leaf development in PHB-producing transgenic sugarcane allows a large flux of carbon to be diverted to PHB synthesis which normally would not be readilly accessible due to substrate competition. In the fully differentiated leaf, where the fatty acid synthesis is lower, its inhibition does not significantly improve PHB production.

### PHB production affects gluconeogenesis and starch biosynthesis

The previously reported absence of starch granules in BS chloroplasts of high PHB producing switchgrass plants [15] suggested that polymer synthesis affects carbon partitioning. The results presented here demonstrated significantly decreased levels of starch and sucrose in high PHB producers (Figure 5) suggesting reduced synthesis rate and/or premature degradation. Glucose levels in sugarcane were also significantly decreased with high levels of PHB production, whereas switchgrass retained its low glucose content independent of polymer synthesis.

Taken together, the experimental data suggests that the PHB producing plants have to readjust carbon fluxes to account for the added sink, and that this adjustment comes at the expense of starch, not fatty acids. Reducing the starch pool, that is likely not vital for growth in C_4_ plants [22] is a preferable strategy for obtaining the carbon needed to supply the novel carbon sink than limiting fatty acid biosynthesis in leaves, which is critical for plant growth. Since the primary location of starch in differentiated leaves of C_4_ plants is BS plastids, this may account, in part, for the difference in PHB synthesis in M and BS cells.

## Conclusions

The low production rate of PHB in M is not due to poor targeting or activity of the PHB biosynthetic pathway in M chloroplasts. Evidence suggests that PHB synthesis may not compete effectively with fatty acid synthesis for substrate in M cells as evidenced by increased polymer formation in M cells following chemical inhibition of ACCase. Although sucrose and starch levels are greatly decreased in high PHB producing plants, redirection of carbon from carbohydrates to PHB is probably not substantial enough to account for this decrease. New strategies are required to increase PHB production in C_4_ grasses to commercially relevant levels. One strategy could be to enhance the ability of the PHB pathway to capture substrate under competitive conditions. A novel acetoactyl-CoA synthase was recently discovered which could fill the same role as the ketothiolase in the PHB pathway and has properties which suggest it may be a superior catalyst for initiation of PHB production [40]. This might help solve the substrate accessibility issue in M plastids. Another strategy could be to increase flux through C_4_ photosynthesis to produce increased levels of polymer while limiting agronomic penalties. We have recently made progress in this area [11] by introducing a cyanobacterial gene encoding a bifunctional enzyme with both fructose-1,6-bisphosphatase (FBPase) and sedoheptulose-1,7-bisphosphatase (SBPase) activities [41,42] into well characterized PHB producing switchgrass lines by re-transformation. Engineering central carbon metabolism is, however, a non-trivial exercise and requires a detailed understanding of the physiology and biochemistry of the plant system at hand. A systems biology approach to PHB biosynthesis in C_4_ crops would provide new information about the regulation and function of the C_4_ and C_3_ photosynthetic pathways in M and BS cells and provide insights on cell-specific strategies to further increase carbon flow to polymer production. This knowledge is critical for optimising plant biofactories for the production of industrial materials, chemicals, and energy.

## Methods

### Plant material

The production and identification of PHB-producing sugarcane (*Saccharum* sp. cv Q117) and switchgrass (*Panicum virgatum* L.) plants used in this study were described previously [12,13,15]. For simplicity, sugarcane and switchgrass lines have been labelled according to the amount of PHB they produce in mature leaves under glasshouse conditions i.e. wild type (WT), low producers (LP, ≤1% DW PHB), medium producers (MP, >1% ≤ 2% DW PHB) and high producers (HP, >2% DW PHB).

### Separation of chloroplasts from mesophyll and bundle sheath cells

M and BS chloroplasts were isolated according to the method described by Majeran et al. [43] with some modifications. For sugarcane, the first leaf with visible dewlap was harvested from four replicates of each sugarcane line tested. In total two to three grams of leaves were collected. Leaves were cut into small pieces and placed in a small Waring blender with 100 mL of cold grinding buffer (50 mM HEPES, pH 7.9, 350 mM sorbitol, 2 mM EDTA, 5 mM ascorbic acid and 5 mM L-cysteine).

Sugarcane leaves were blended twice (ten second pulse each) on a low setting mode. Released M chloroplasts were filtered through 20 μm nylon mesh (Small Parts Inc., Miramar, FL, USA) and stored on ice while BS chloroplasts were extracted. The residual M attached to BS strands were removed by two or more 30-second pulses on high setting in a small Waring blender with the blade reversed, followed by washing with grinding buffer. Chloroplasts were released from purified BS fibres by blending on high setting, in a small Waring blender with the blades replaced with a razor blade, in 30 second pulses until the grinding buffer turned green. BS chloroplasts were filtered through 20 μm nylon mesh to remove cellular debris. After centrifugation at 1000 g for four minutes, chloroplasts from both fractions were precipitated. Pure chloroplasts were observed after additional washing with grinding buffer and centrifugation.

For isolation of chloroplasts from switchgrass, the second leaf from the base of vegetative tillers at an early elongation stage (three to four tillers per line) from transgenic and wild type plants were harvested. The leaves were cut into small pieces and gently ground by mortar and pestle in the buffer described above. Released M chloroplasts were collected and stored as described for sugarcane. The residual M cells attached to BS strands were removed by vigorous grinding followed by washing. The purity of the BS strands was assessed by microscopy at this step. BS chloroplasts were released by further grinding then passed through a 20 μm nylon mesh. Chloroplasts from both fractions were pelleted and washed as described above for sugarcane.

### Enzyme assays

PhaA was assayed in the thiolysis direction using the procedure described by Palmer et al. [44] with some modifications. The assay mixture (1 mL) contained 62.4 mM Tris-Cl pH 8.1, 50 mM MgCl_2_, 62.5 μM CoA, and 62.5 μM acetoacetyl-CoA. The loss of acetoacetyl-CoA was monitored with time (ϵ304 = 16.9 × 10^3^ cm^−1^ M^−1^). The reaction was initiated by addition of the total protein extract and the absorbance at 304 nm was monitored at 25°C.

PhaB was assayed in the direction of 3-hydroxybutyryl-CoA formation by NADPH reduction using the procedure described by Ploux et al. [45] with modifications. The assay mixture (1 mL) contained 100 mM Tris-Cl pH 8.1, 100 μM NADPH, and 20 μM acetoacetyl-CoA. The loss of NADPH was monitored with time (ϵ340 = 6.22 × 10^3^ cm^−1^ M^−1^). The reaction was initiated by addition of the total protein extract and the absorbance at 340 nm was monitored (25°C). Protein concentrations were determined using Bradford reagent (Sigma-Aldrich). The specific enzyme activity of each sample was calculated as units of enzyme per mg of total protein where one unit is defined as the amount of enzyme that catalyses the transformation of 1 μmol substrate/min.

### Immunolocalization

Leaf sections were prepared for immunofluorescence microscopy using the procedure described by Paciorek et al. [46]. Thin transverse leaf sections (6–7 μm) were cut and labelled with primary rabbit antisera specific to *Ralstonia eutropha* β-ketothiolase (1:10000 dilution) or acetoacetyl-CoA reductase (1:5000 dilution) as described above for protein immunoblots. Alexafluor 488 goat anti-rabbit IgG (Invitrogen) was used as a secondary antibody (1:5000 dilution). Images were acquired using a Zeiss LSM 510 META confocal microscope (488 nm excitation; BP 500–550 nm).

### PHB analyses

Approximately 10 mg of sugarcane leaf material was analysed for PHB content by HPLC following acid digestion to crotonic acid as previously described in Petrasovits et al. [12]. For switchgrass, 20–80 mg of lyophilized leaf tissue was prepared for analysis by gas chromatography/mass spectrometry (GC/MS) using a simultaneous extraction and butanolysis procedure as described by Kourtz et al. [47].

### *In vivo* ACCase inhibition assay

ACCase inhibitor solution was prepared by diluting the commercially available herbicide Fusilade Forte (128 g L^−1^ of active ingredient fluazifop-P; Syngenta) in distilled water containing Searles Spreadmax wetting agent (700 μl L^−1^). Approximately 5 mL of 20 μM or 100 μM herbicide solution was applied to the leaves of five week old sugarcane plants grown in a glasshouse. Treated plants were grown for an additional 14 days. All fully expanded leaves were collected separately according to leaf number, dried and analysed for PHB content.

Switchgrass plants were propagated from PHB producing plants following a procedure described previously [11] and grown under *in vitro* conditions. Briefly, immature inflorescence-derived callus cultures were initiated from soil-grown primary transformants producing >2% DW PHB). Plants regenerated from these cultures on MS basal medium supplemented with 30 g L^−1^ maltose and 1.4 μM gibberellic acid [48] were grown at 27°C, with a 16 h photoperiod (cool white fluorescent bulbs, 80 μmol m^−2^ s^−1^) for about six weeks. Two weeks prior to treatment, plantlets with four to five leaves were transferred to larger tissue culture containers (5 plants per container) and grown under the conditions described above. Switchgrass plantlets were sprayed with approximately 900 μl of quizalofop solution. The herbicide solution was prepared with 90% ethanol (v/v) and diluted to final concentrations of 0.05, 0.10, 50, and 100 μM. Wild-type plantlets treated with 50 nM quizalofop or 90% ethanol (v/v) as well as PHB producing plantlets treated with 90% ethanol (v/v) served as controls. Ten days after the herbicide treatment, leaf segments were stained with Nile Blue A as previously described [28] for visualization of the polymer granules. For measurements of the total fatty acids and polymer contents, whole plantlets without roots were harvested (five plants per treatment) and freeze-dried for three days prior to analyses. At these early stages of their growth under *in vitro* conditions, plants have developing leaves and no differentiated stem tissues.

### Starch, sucrose and glucose measurements

Transgenic and wild type plants were grown in a glasshouse under similar light intensity. Sugarcane plants were grown from setts for approximately four months and the first fully unfurled leaf was harvested (four plants per line). Switchgrass plants were grown for about four months after re-potting and the leaf adjacent to the second node from the base of vegetative tillers at an early elongation stage (three tillers per plant) was sampled. Both sugarcane and switchgrass samples were collected between midday and 1 pm on a sunny day and snap frozen in liquid nitrogen. The leaves were freeze-dried, ground to a fine powder and used for analysis of starch, soluble sugars and PHB. PHB content was determined using the methods described above. For measurements of starch content, 50 mg of tissue was extracted with 80% ethanol to remove soluble sugars. The percentage of starch per dry weight was measured as glucose, following digestion with α-amylase and amyloglucosidase using the starch (GO/P) assay kit (Sigma, STA20) according to the manufacturer’s instructions. For analysis of soluble sugars, 20–50 mg of sample was extracted with 1 mL of hot water. The extract was analysed by HPLC using a Shodex KS-801 column (Tosho) and a refractive index detector. A column temperature of 80°C and a flow rate of 1 mL/min were used.

### Fatty acid content

The amount of total fatty acids in leaf tissue from switchgrass plants was determined by converting fatty acids to their methyl esters and measuring the content by gas chromatography. Methyl esters of the following fatty acids were analysed: palmitic (C16:0), stearic (C18:0), oleic (C18:1), linoleic (C18:2), linolenic (C18:3), arachidic (C20:0), eicosanoic (C20:1), eicosadienoic (C20:2), eicosatrienoic (C20:3), behenic (C22:0), and erucic acid (C22:1). Published procedures were followed [49] with modifications. Briefly, freeze-dried leaf tissue (30 to 50 mg) was placed in screw-cap test tubes and mixed with 1.5 ml of 2.5% (v/v) sulfuric acid in methanol (w/ 0.01% w/v BHT), 400 μl toluene, and 500 μg of a triheptadecanoin (Nu-Chek Prep, Elysian, MN) solution (10 mg/ml in toluene) as internal standard. The tubes were purged with nitrogen, capped, and heated at 90°C for 1 h. Upon cooling, 1 ml of 1 M sodium chloride and 1 ml of heptane were added to each tube. Following mixing and centrifugation, the heptane layer containing fatty acid methyl esters was analysed with an Agilent 7890A gas chromatograph. Fatty acid methyl esters were resolved with a 30 m × 0.25 mm (inner diameter) INNOWax column (Agilent) and detected by flame ionization. The oven temperature was programmed from 185°C (1 min hold) to 235°C (1 min hold) at a rate of 10°C/min (11 min total run time), and the front inlet pressure was 35.8 psi of He. The total fatty acid content was determined by comparison of the detector response from leaf-derived fatty acid methyl esters relative to methyl heptadecanoate from the triheptadecanoin internal standard.

### Statistical analysis

Statistical analyses were performed using SigmaPlot for Windows Version 11.0 (Systat Software, Inc.). Multiple comparisons were done by one way ANOVA using the default suggested test at P < 0.001.

## Abbreviations

ACCase: Acetyl-CoA carboxylase; BS: Bundle sheath; CO2: Carbon dioxide; DW: Dry weight; FAS: Fatty acid synthesis; HP: High producer; PHB: Polyhydroxybutyrate; LP: Low producer; M: Mesophyll; MP: Medium producer; NAD-ME: NAD dependent malic enzyme; NADP-ME: NADP-dependent malic enzyme; PHAs: Polyhydroxyalkanoates; PHB: Polyhydroxybutyrate; Rubisco: Ribulose-1,5-bisphosphate carboxylase/oxygenase; WT: Wild-type.

## Competing interests

MNS and KDS are employees of Metabolix, Inc. XL is a former employee of Metabolix, Inc.

## Authors’ contributions

RBM, LAP, LKG, MNS, KDS, XL, LKN and SMB contributed to research design. RBM, LKG, XL, and MNS performed experiments; RBM performed statistical analyses. RBM, LAP, LKG, MNS, and KDS wrote the paper. All authors discussed the results and commented on the manuscript. All authors read and approved the final manuscript.
